# Microgel Particles with Distinct Morphologies and Common Chemical Compositions: A Unified Description of the Responsivity to Temperature and Osmotic Stress

**DOI:** 10.3390/gels6040034

**Published:** 2020-10-16

**Authors:** Andrea Ruscito, Ester Chiessi, Yosra Toumia, Letizia Oddo, Fabio Domenici, Gaio Paradossi

**Affiliations:** Department of Chemical Sciences and Technologies, University of Rome Tor Vergata, Via della Ricerca Scientifica I, 00133 Roma, Italy; andrea.ruscito93@gmail.com (A.R.); ester.chiessi@uniroma2.it (E.C.); Yosra.Toumia@uniroma2.it (Y.T.); letizia.oddo@uniroma2.it (L.O.); fabio.domenici@uniroma2.it (F.D.)

**Keywords:** PNIPAM, hydrogels, drug delivery systems, Flory–Rehner, swelling, osmotic pressure, crosslinks

## Abstract

Poly(N-isopropylacrylamide) (PNIPAM) hydrogel microparticles with different core–shell morphologies have been designed, while maintaining an unvaried chemical composition: a morphology with (i) an un-crosslinked core with a crosslinked shell of PNIPAM chains and (ii) PNIPAM chains crosslinked to form the core with a shell consisting of tethered un-crosslinked PNIPAM chains to the core. Both morphologies with two different degrees of crosslinking have been assessed by confocal microscopy and tested with respect to their temperature responsivity and deformation by applying an osmotic stress. The thermal and mechanical behavior of these architectures have been framed within a Flory–Rehner modified model in order to describe the microgel volume shrinking occurring as response to a temperature increase or an osmotic perturbation. This study provides a background for assessing to what extent the mechanical features of the microgel particle surface affect the interactions occurring at the interface of a microgel particle with a cell, in addition to the already know ligand/receptor interaction. These results have direct implications in triggering a limited phagocytosis of microdevices designed as injectable drug delivery systems.

## 1. Introduction

Responsive hydrogels are attracting the attention of researchers working on biosensors, drug delivery, and micromotors [1,2,3]. One of the most investigated classes of responsive hydrogels is based on the polymerization and crosslinking of N-isopropylacrylamide (NIPAM) monomers. Such hydrogels display a volume phase transition (VPT) at temperatures close to the physiological one, leading to a number of biomedical applications. The scaling down of monolithic, wall-to-wall hydrogels to micron and nano-sized gel particles without losing the specific responsivity [4] has offered the possibility to formulate biomedical micro- or nano-devices able to reach the tissue or organ to be treated. Many chemical and structural variants of microgels based on poly(N-isopropylacrylamide) (PNIPAM) have been synthesized, including co-monomers to add other kinds of responsivity [5], obtaining microgels with uniform or inverted polymer distribution [3,6,7,8]. With the careful use of classic polymerization methods, such as precipitation polymerization, new architectures with responsivity to temperature and with controlled rigidity, but maintaining the same chemical composition, can be obtained, allowing to test the microgels’ mechanical features with respect to phagocytosis or other processes having the effect to bring cells in close contact with exogenous microparticles. The molecular recognition pathways based on receptor/ligand binding do not exhaust the factors affecting the behavior of cells with respect to the stiffness of a particle surface [9]. Information about this feature can be obtained by osmotic stress tests, in connection with different particle morphologies. In this respect, the bulk modulus of PNIPAM microgels copolymerized with poly(ethylene glycol) diacrylate has been determined by measuring the volume deformation of the microgel as a function of osmotic pressure using dextran solutions [10,11], and with similar approaches the micromechanics of polymer microparticles can be accessed [12]. The importance of the osmotic stimulus can be easily understood if we consider that injected microgel particles are subject to blood and osmolyte pressure, which may influence the size and stiffness of the particles. Consequently, the volume phase transition occurring with a temperature-driven volume shrinking, in the case of PNIPAM networks, can be triggered also by a mechanical stimulus such as an osmotic stress.

In this study we investigated two types of PNIPAM microgel particles with distinct architectures obtained by precipitation polymerization: a core-shell (CS) type and a dense core (DC) type of microgel particle. In the CS type, the polymer chains of the core are not cross-linked, unless for the presence of autocrosslinks introduced during this type of polymerization, while the shell is crosslinked. In the DC type, the crosslinked polymer core has a corona of dangling chains with no crosslinks. The crosslinked domains of these two architectures have been obtained at two degree of crosslinks, i.e., 0.1 and 0.5%, respectively.

The different morphologies were studied during the gel collapse activated by a temperature increase or an osmotic pressure build up. In particular we address whether it is possible to qualitatively unify the dependence of the volume phase transition from temperature and osmotic stress within the frame of the classic Flory-Rehner “elastic polymer solution” model. A further development of this investigation will be the quantitative assessment of phagocytosis when macrophages interact with the gel particles with different morphologies and rigidity. This is a key point in modulating the primary immune response in order to limit the phagocytosis of microgels injected in the blood stream as drug carriers.

## 2. Results and Discussion

### 2.1. Morphological Characterization of PNIPAM Microgels

**Scheme 1:**
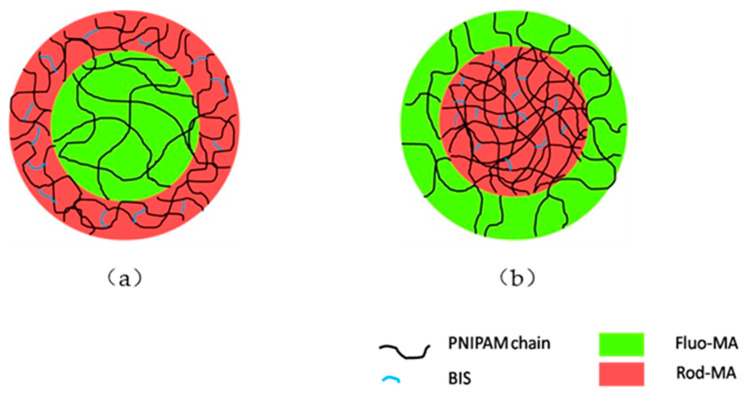
Schematic of the two types of PNIPAM microgels in a swollen state with different spatial cross-linking density distributions, prepared by surfactant-free precipitation polymerization: (**a**) microgels with an un-crosslinked core and a crosslinked shell (CS); (**b**) microgels with a crosslinked core and an un-crosslinked periphery (DC). Green and red highlighted regions refer to the labeling of microgel domains with Fluo-MA and Rhod-MA, respectively (see text for details).

In line with our previous investigations on hybrid microgels based on PNIPAM networked with hyaluronic acid via click chemistry [13,14], in this work we addressed the importance of the microgel architecture, maintaining the chemical composition of the network unvaried. We synthesized PNIPAM microgels having two distinct morphologies characterized by different spatial distribution of crosslinking density (Scheme 1). Temperature-controlled surfactant-free precipitation polymerization [15], carried out in semi-batch mode, allowed for the preparation of microgel particles with an un-crosslinked core and a crosslinked shell (CS; Scheme 1a) or, alternatively, particles with a crosslinked core and an un-crosslinked periphery (DC; Scheme 1b). Furthermore, for each morphology, two degree of crosslinking were probed by using a % feed molar ratio of N,N’-methylenebisacrylamide (BIS) to a total amount of NIPAM monomer of 0.5% mol/mol (CS_05 and DC_05) and 0.1% mol/mol (CS_01 and DC_01).

The colloidal stability of the microgel particles is generally ensured by electrostatic stabilization due to sulfate groups derived from the initiation step and remaining as chain ends after the polymerization [16]. In the case of the DC type, the microgel stability is also provided by the steric contribution of the free chains hanging out from the particle dense core.

It is known and well documented that during the networking by free radical polymerization, the crosslinks are not uniformly distributed in nanocompartmentalized microgel particles [17]. Super high-resolution microscopy has allowed uneven distribution to be detected [18,19] in the nanoscopic spatial range, highlighting a higher chain density toward the center of the particle core. However, for low crosslinking degrees, this distribution gradient is counterbalanced by the simultaneous diffusion-driven action of the potassium persulfate (KPS) redox initiator, consisting of a hydrogen abstracting effect, which increases the number of crosslinks in the periphery of the particle [6]. How these complex crosslinking patterns reflect on the architectures of our microgel particles is a difficult issue to solve.

**Figure 1:**
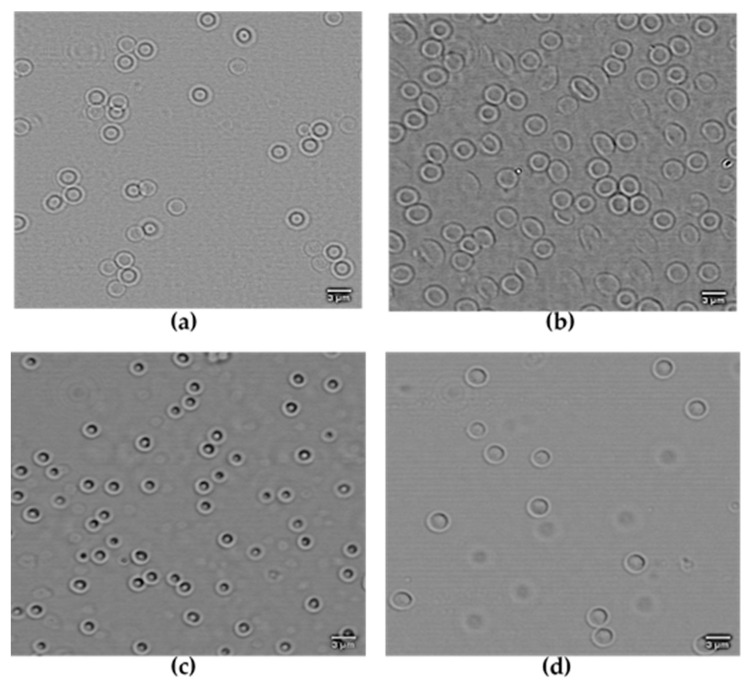
Optical microscope images of PNIPAM microgels in deionized water at room temperature: (**a**) CS_05 microgels; (**b**) CS_01 microgels; (**c**) DC_05 microgels; (**d**) DC_01 microgels. Scale bar = 3 µm.

In the CS architecture, comparable concentrations of BIS crosslinker and KPS should yield negative and positive competing density gradients, respectively, compensating each other [6] within a shell thickness of a few hundred nanometers. The core of this type of microgel particle does not contain BIS crosslinker; however, we cannot exclude the existence of a thin outermost ring of crosslinks merging with the crosslinked shell, due to the diffusion of KPS. The DC microgels, in analogy to the reported examples in the literature [20], will have a core with a decreasing crosslink density radial gradient, contrasted by an opposite chain density gradient due to the proton abstraction effect of KPS, but distinct from the fuzzy crown made of freely dangling chains, propagating in the absence of crosslinker, although containing some autocrosslinked units. This picture is qualitatively in agreement with the differential interference contrast (DIC) optical microscopy images of the four types of PNIPAM microgels prepared, after overnight equilibration of the dried microgels in deionized water at room temperature, shown in Figure 1. A ring structure is common to all types of microgels with similar average diameter lower than 3 µm. CS_01, as seen in Figure 1b, appears to have a higher deformability and a more contrasted shell.

Furthermore, microgel particles DC_05, as seen in Figure 1c, display a smaller (≤1 μm) core than the other types. However, as it is shown below by the Dynamic Light Scattering (DLS) analysis, these particles have overall dimension comparable to the others (around 3 μm).

**Figure 2:**
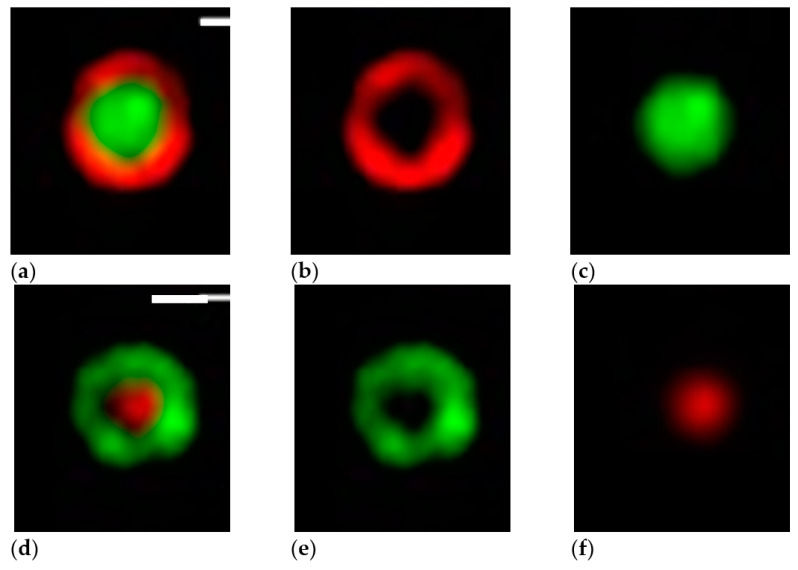
Representative confocal laser scanning microscopy (CLSM) images of fluorescent PNIPAM microgels in deionized water at room temperature: (**a**) CS_05 microgels, fluoresceine and rhodamine merged channels; (**b**) CS_05 microgels: crosslinked shell labeled with Rhod-MA; (**c**) CS_05 microgels: un-crosslinked core labeled with Fluo-MA; (**d**) DC_05 microgels, merged channels; (**e**) DC_05 microgels: un-crosslinked shell labeled with Fluo-MA; (**f**) DC_05 microgels: crosslinked core labeled with Rhod-MA. Scale bar = 1 μm.

**Figure 3:**
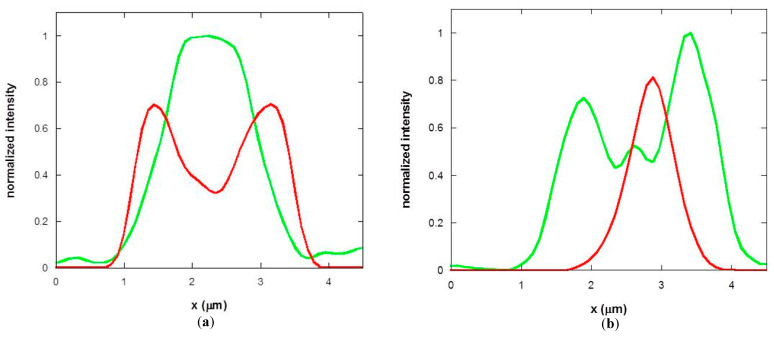
Representative intensity profiles of fluorescent PNIPAM microgels in deionized water at room temperature. Green solid line: intensity profile relative to Fluo-MA; red solid line: intensity profile relative to Rhod-MA. (**a**) CS_05 microgels, green: core; red: shell; (**b**) DC_05 microgels, green: shell; red: core.

In order to gain more insight on CS and DC microgel morphology, we specifically labeled the two domains of the particles, namely the core and the shell, with two fluorescent probes: fluorescein O-methacrylate (Fluo-MA; see Figure S1 of the Supplementary Materials) and methacryloxyethyl thiocarbamoyl rhodamine B (Rhod-MA; see Figure S2 of the Supplementary Materials). These fluorescent dyes, due to the vinyl moiety contained in the methacrylate ester functional group, could be copolymerized with NIPAM and BIS during microgel synthesis. In particular, Rhod-MA was used to label the region of the microgels where PNIPAM chains were crosslinked with BIS (shell of CS microgels and core of DC microgels), while Fluo-MA was used to highlight the un-crosslinked domains of the particles (core of CS microgels and shell of DC microgels). In order to avoid any perturbations in the particle structure, dyes were used in very small amounts, i.e., 0.003% mol/mol of Rhod-MA and 0.008% mol/mol of Fluo-MA to NIPAM monomer added for the core or the shell synthesis. According to a preliminary optimization study on the synthesis of fluorescent PNIPAM microgels (not reported here), the polymerization reaction was carried out at lower temperature and for a shorter time (50 min at 60 °C) as compared to the procedure followed for non-fluorescent microgels (4 h at 70 °C); furthermore, microgels were also immediately purified after synthesis. This protocol was adopted to avoid the massive precipitation of clustered polymeric materials and the loss of the fluorescence of the probes, especially of the Fluo-MA. The scanning of the focused equatorial plane of fluorescent microgels (Figure 2) and the analysis of the fluorescence intensity spatial profiles (Figure 3), measured across the microparticle, allowed the evaluation of the average outer diameters of the fluorescent microgels CS_05 and DC_05, which were found to be 2.4 ± 0.4 μm and 2.5 ± 0.4 μm, respectively. Figure 3b confirms the observation, gathered from DIC microscopy, that the core size of DC_05 is smaller than in the other morphologies. Overall the distance between the center of the particles and its periphery is around 1 µm, too small to collect many z-plan confocal images with the instrument resolution along z-axis of around 0.6 µm. The confocal microscopy images were taken without anchoring the microgel particles to a solid substrate avoiding any shape perturbation [21], but waiting long enough to let the particles sediment on the glass slip. This appeared to be enough to avoid image smearing due to the diffusion of microparticles with an overall size of 2–3 µm. From fluorescence intensity profiles, it was also possible to extrapolate the average diameter of the core and the thickness of the microgel particle peripheries. However, since these values are close to the limit of the optical resolution (~180 nm) provided by confocal microscopy, an accurate measure of them requires a complementary technique [22,23]. Under these assumptions, this analysis provided a mean core diameter of 1.4 ± 0.3 μm for fluorescent microgels CS_05 and of 0.8 ± 0.2 μm for fluorescent microgels DC_05; while the thicknesses of the particle peripheries were estimated to be 0.5 ± 0.1 μm and 0.8 ± 0.1 μm for fluorescent microgels CS_05 and DC_05, respectively.

These values roughly reflect the spatial distribution of crosslinked domains within the microgels structures, with a more compact region where the PNIPAM chains are crosslinked. Furthermore, it should be considered that in the case of DC microgels, the amount of BIS was added all at once at the beginning of the synthesis; also, it is known that during the particle formation, the BIS is incorporated into the microgel structure faster compared to the NIPAM monomer, leading to the so-called “core-fuzzy shell” structure of microgels [24,25]. From these considerations, it cannot be excluded that also the core of fluorescent DC_05 microgels is characterized by a crosslink density gradient with a mostly cross-linked internal region with a diameter smaller than 0.8 ± 0.2 μm, although the counteracting KPS autocrosslinking attenuates this effect.

**Figure 4:**
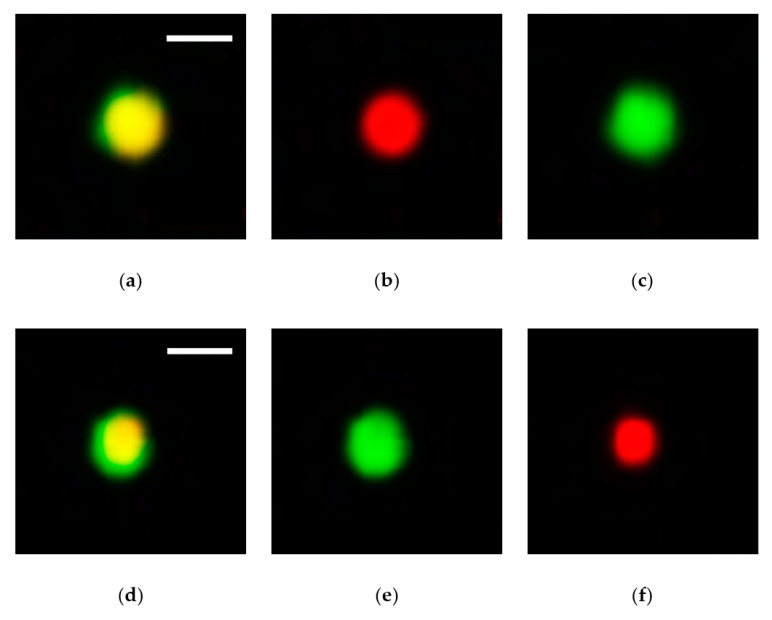
Representative CLSM images of deswelled fluorescent PNIPAM microgels in PEG (*M_n_* 4000, 19.2% *w*/*v*, osmotic pressure = 1.2 atm) at room temperature: (**a**) CS_05 microgels, merged channels; (**b**) CS_05 microgels: crosslinked shell labeled with Rhod-MA; (**c**) CS_05 microgels: un-crosslinked core labeled with Fluo-MA; (**d**) DC_05 microgels, merged channels; (**e**) DC_05 microgels: un-crosslinked shell labeled with Fluo-MA; (**f**) DC_05 microgels: crosslinked core labeled with Rhod-MA. Scale bar = 1 μm.

**Figure 5:**
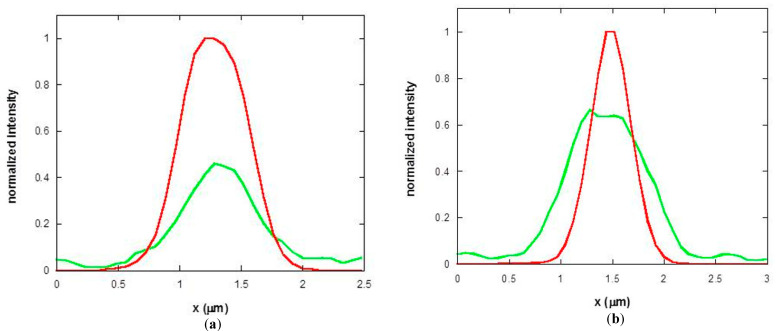
Representative intensity profiles of deswelled fluorescent PNIPAM microgels in PEG (*M_n_* 4000, 19.2% *w*/*v*, osmotic pressure = 1.2 atm at room temperature). Green solid line: intensity profile relative to Fluo-MA; red solid line: intensity profile relative to Rhod-MA. (**a**) CS_05 microgels, green: core; red: shell; (**b**) DC_05 microgels, green: shell; red: core.

To monitor the action of an external osmotic stress on the synthesized structures, we visualized the deswelling of the fluorescent microgel particles upon addition in the aqueous medium of polyethyleneglycol (PEG) *M_n_* 4000, 19.2% *w*/*v*, which, assuming an ideal solution behavior, exerts an osmotic pressure of 1.2 atm at room temperature. As shown in Figure 4, under these conditions the microparticles shrunk, and the structural details fell below the resolution of the microscope. As is evident from the images, in the collapsed state, the ring structure of the microparticle shell is s no longer visible in both microgel morphologies (Figure 4 and Figure 5). This can be explained considering the Rayleigh criteria for the resolution. With this criteria, the sum intensity of two close objects (represented by two opposite edges of the shell across the microparticle) should have an intensity minimum that is 20–27% lower than the peak intensity [23]. This condition is no longer satisfied when the microgels are in the collapsed state. However, it is possible to highlight a difference between the two deswelled morphologies; in fluorescent DC_05 microgels the core of the particles is confined well inside the deswelled microparticles, with a collapsed core average diameter around 0.4 μm. In fluorescent CS_05 microgels, on the other hand, the boundaries of the core and the shell are almost completely superimposable.

Overall diameters of 0.7 ± 0.1 μm and 0.8 ± 0.1 μm were estimated from the fluorescence profile of microgels CS_05 and DC_05, respectively (Figure 5), using the larger half width at half height of the peak traces.

### 2.2. Determination of the Maximum Swelling Degree of PNIPAM Microgels

Several attempts to determine the water content of microgel microparticles are described in the literature [26,27,28,29,30]. However with these methods it is difficult to discriminate between water that is caged in the microparticles and interstitial free water present between the microspheres, leading to results with low accuracy. Hence, in this work we adopted an approach, derived by the method developed by Stenekes and Hennink [31,32], exploiting the effect of the hydration process of lyophilized dried microgels on an aqueous solution of fluorescein tagged PEG, *M_n_* 4000 (FITC-PEG). The use of PEG was suitable for this aim due to the lack of interaction with PNIPAM [33], and the fluorescence tagging allowed a very low concentration of polymer (2 µg/mL) and a negligible osmotic effect, not perturbing the swelling conditions of the microgel. Furthermore, PEG dimensions were such to exclude the polymer chains from the PNIPAM microgel meshes, as demonstrated by confocal microscopy (not shown). Consistently, the osmotic pressure effect exerted by higher PEG concentrations (8 mg/mL and up) on microgel size, shown by CLSM (Figure 4) and by dynamic light scattering below, were completely reversible after PEG removal from the microgels suspensions by centrifugation, indicating that the polymer could not freely enter the microgel particles (see Table S1, Supplementary Materials)

With this method, water is absorbed by the dried microparticles, and consequently the concentration of FITC-PEG in solution increased; this increase in the supernatant of the microgel suspensions could be easily quantified by fluorescence spectroscopy and related to the amount of water absorbed by the microgels. Hence, from the amount of the equilibrium water content, the water weight fraction over the microgel total weight and the polymer volume fraction (*ϕ*) were derived according to Equation (1):

(1) $$\phi =\frac{\frac{w_{p}}{d_{p}}}{\frac{w_{p}}{d_{p}}+\frac{w_{H2O}}{d_{H2O}}}=\frac{1}{\left[1+\frac{d_{p}}{d_{H2O}}\left(\frac{1-w_{p}}{w_{p}}\right)\right]}$$

where *w_p_* is the weight fraction of PNIPAM, *w_H_*_2*O*_ the weight fraction of water, *d_p_* is PNIPAM density of the dry polymer, i.e., 1.1 g/mL [34], and *d_H_*_2*O*_ is the density of water. With the density of water of 1 g/mL, Equation (1) becomes

(2) $$\phi =\frac{1}{1+\frac{\left(1-w_{p}\right)}{w_{p}}\;1.1}$$

The values obtained are reported in Table 1.

### 2.3. Dependence of PNIPAM Microgel Size on Temperature and Osmotic Stress

**Figure 6:**
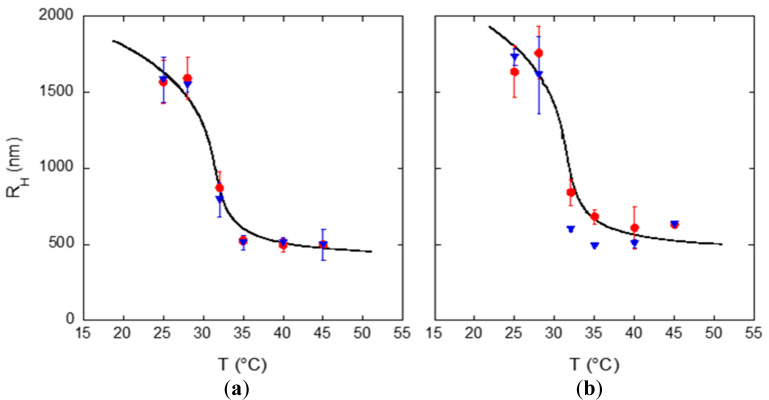
Temperature behavior of the average hydrodynamic radius (*R_H_*) of CS_05 (**a**) and CS_01 (**b**) as obtained from DLS measurements in water. Red and blue symbols refer to heating and cooling scan, respectively. The black line is the fitting curve with the Flory–Rehner model (see text). Error bars represent the standard deviation on three independent experiments.

**Figure 7:**
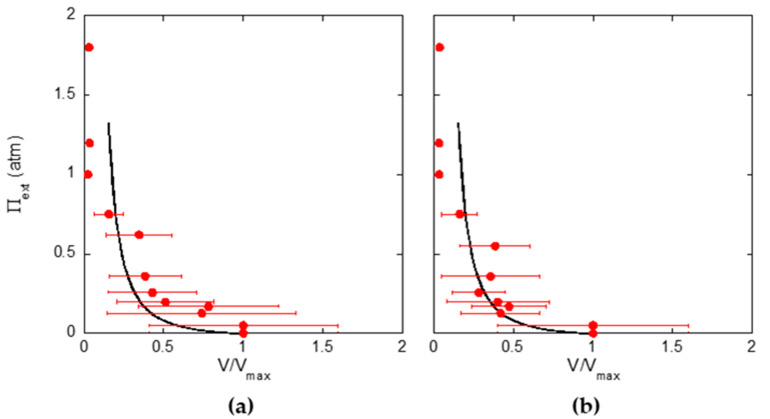
Correlation between the osmotic pressure of the external solution (*Π_EXT_*) and the shrinking ratio (*V*/*V_max_*) at 25 °C for CS_05 (**a**) and CS_01 (**b**) microgels, as obtained from DLS measurements in aqueous solutions of PEG. The black line is the fitting curve with the Flory–Rehner model (see text). Error of volume ratio is obtained by the propagation of *R_H_* error, estimated as the standard deviation on three independent DLS experiments.

**Figure 8:**
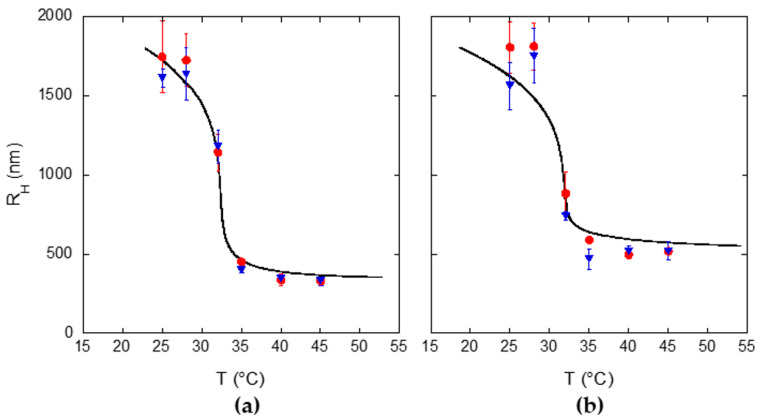
Temperature behavior of the average hydrodynamic radius of DC_05 (**a**) and DC_01 (**b**) as obtained from DLS measurements in water. Red and blue symbols refer to heating and cooling scan, respectively. The black line is the fitting curve with the Flory–Rehner model (see text). Error bars represent the standard deviation on three independent experiments.

**Figure 9:**
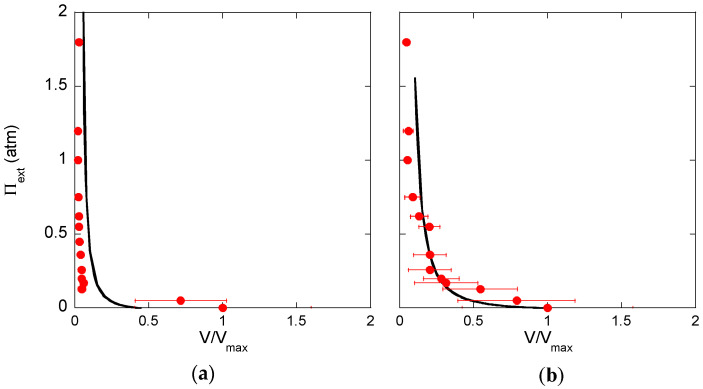
Correlation between the osmotic pressure of the external solution (*Π_EXT_*) and the shrinking ratio (*V/V_max_)* at 25 °C for DC_05 (**a**) and DC_01 (**b**) microgels, as obtained from DLS measurements in aqueous solutions of PEG. The black line is the fitting curve with the Flory–Rehner model (see text). Error of volume ratio is obtained by the propagation of *R_H_* error, estimated as the standard deviation on three independent DLS experiments.

We monitored the behavior of the size of microgel particles in aqueous diluted suspension as a function of temperature in the interval 25–45 °C (Figures 6 and 8) and as a function of the osmotic regime at 25 °C (Figures 7 and 9), and the results were framed in the Flory–Rehner model of the “elastic polymer solution” [35]. This approach to the description of microgel particles differing from each other by the spatial distribution of the matter, allowing the chemical composition of the network to be unchanged, seemed the most suitable to determine the thermodynamic potentials acting on their overall behavior. Atomistic approaches to model small parts of the microgel, already used by us for the polymer in aqueous solution with a good agreement to the experimental thermoresponsivity and hydration state at molecular level [36,37], were considered not adequate for a large scale description of the responsivity of microgels.

According to the Flory–Rehner model, the osmotic pressure (*Π*) within the particle is given by the sum of an elastic and a mixing contribution, *Π_ELASTIC_* and *Π_MIX_*, respectively, both terms dependent on the polymer volume fraction in the microgel (*ϕ*):

(3) $$\Pi \left(\phi \right)=\Pi {\left(\phi \right)}_{MIX}+\Pi {\left(\phi \right)}_{ELASTIC}$$

The mixing term is illustrated in Equation (4):

(4) $$\Pi_{MIX}\left(\phi \right)=\frac{k_{B}T}{a^{3}}\left\{-\phi -\ln\left(1-\phi \right)-\chi \phi^{2}\right\}$$

where *a*^3^ is the molecular volume of the solvent and *χ* is the Flory–Huggins solubility parameter. The dependence of *χ* on temperature and concentration [38] is accounted as

(5) $$\chi =0.5-A\left(1-\theta /T\right)+C\phi +D\phi^{2}$$

where *θ* is the theta temperature, *C* and *D* are coefficients independent on temperature and *A* is related to the non-combinatorial entropy change for the transfer of a polymer segment from bulk polymer to the solution (*ΔS*), as in Equation (6):

(6) $$A=-\frac{1}{2}\left(\frac{\Delta S}{k_{B}}-1\right)$$

The elastic term is formulated assuming an affine network:

(7) $$\Pi {\left(\phi \right)}_{ELASTIC}=\frac{N_{c}k_{B}T}{V_{0}}\left[\frac{1}{2}\left(\frac{\phi }{\phi_{0}}\right)-{\left(\frac{\phi }{\phi_{0}}\right)}^{1/3}\right]$$

where *N_c_* is the number of chains in the microgel network, *V_0_* and *ϕ_0_* are the volume and the polymer volume fraction of the microgel in the reference state, respectively. The expression of *Π_ELASTIC_* can be rearranged to explicitly include the degree of cross-linking, f, equal to the molar ratio of cross-linker to the sum of monomer and cross-linker, obtaining Equation (8).

(8) $$\Pi {\left(\phi \right)}_{ELASTIC}=k_{B}T\left\{\frac{2\phi_{0}d_{P}f}{M_{0}}\left[\frac{1}{2}\left(\frac{\phi }{\phi_{0}}\right)-{\left(\frac{\phi }{\phi_{0}}\right)}^{1/3}\right]\right\}$$

The equilibrium degree of swelling of the microgel in the aqueous suspension can be calculated by imposing *Π* = 0 in Equation (3). Similarly, when the microgels are equilibrated against an aqueous solution containing an osmolyte unable to permeate the microgel, the degree of swelling can be predicted again by Equation (3), but with *Π* = *Π_EXT_*, namely the value of osmotic pressure of the external medium. In a few studies the Flory–Rehner model is applied to describe the response of neutral microgels to an osmotic stress [39,40].

Figure 6a,b show the behavior as a function of temperature of the hydrodynamic radius (*R_H_*) of microgels CS_05 and CS_01, respectively, for the microgel suspension in water (data reported in Table S2, Supplementary Materials). The sharp decrease of *R_H_*, detected at about 30 °C, is the signature of the VPT from a swollen to a collapsed state of the microgel.

The fitting curves obtained using the Flory–Rehner model are also reported in Figure 6. To calculate these curves, the equilibrium polymer volume fraction, deduced from Equation (1) with *Π* = 0, is converted to the corresponding *R_H_* value by considering that

(9) $${\left(\frac{R_{H}}{R_{H,collapsed}}\right)}^{3}=\frac{\phi_{collapsed}}{\phi }$$

with *R_H,collapsed_* and *ϕ_collapsed_* being the hydrodynamic radius and the polymer volume fraction, respectively, at 40 °C, a temperature above the volume phase transition temperature (VPTT) and where the microgels are in the collapsed state. *R_H,collapsed_* is experimentally determined, whilst the value of *ϕ_collapsed_* was initially estimated by Equation (10).

(10) $${\left(\frac{R_{H}\left(298\;K\right)}{R_{H,collapsed}}\right)}^{3}=\frac{\phi_{collapsed}}{\phi \left(298\;K\right)}$$

where both *R_H_*(298 K) and *ϕ*(298 K), the hydrodynamic radius and polymer volume fraction at 298 K, respectively, are experimental values.

The Flory–Rehner model was able to represent the dependence of *R_H_* on temperature in water with the same set of parameters for both CS_05 and CS_01, except for the value of *ϕ_collapsed_*, which was slightly higher for CS_05 microgel (see Table 2). In particular, the value of f was 0.005, irrespective of the different content of BIS in the microgel syntheses.

Figure 7 illustrates the behavior of the size of CS_05 and CS_01 microgels at 25 °C in aqueous solutions at different concentrations of PEG, corresponding to osmotic pressures in the range 0.05–1.8 atm at room temperature. In particular, Figure 7 shows the correlation of the ratio between the microgel volume (*V*) and the microgel volume in the absence of osmolyte (*V_max_*), in the presence of osmotic pressure from the solution where microparticles were suspended (data reported in Table S3, Supplementary Materials). Microgel volumes were calculated from the average hydrodynamic radii obtained by DLS experiments (Section 4.9). At increasing *Π_EXT_*, the volume of the microgel decreased, up to the 2.3 ± 0.9% and the 3 ± 2% of the volume measured at *Π_EXT_* = 0, for CS_05 and CS_01 microgels, respectively (see Table S3).

It is noteworthy that the extent of the microgel shrinkage induced by the osmotic stress is comparable to that induced by temperature, as reported previously [41]. However, the PNIPAM microgels investigated in that report were characterized by a higher degree of crosslinking and a lower size at 25 °C, as compared to our microgels. In those conditions [41], the minimum value of the deswelling ratio (*V/V_Max_*) was about 0.2, an order of magnitude larger than the value observed for our microgels at the same experimental conditions.

We used the Flory–Rehner model (Equations (3)–(5),(7) and (8)) also to fit the behaviors of Figure 7, by correlating the *V*/*V_max_* ratio to the polymer volume fraction by means of Equation (11):

(11) $$\frac{V}{V_{max}}=\frac{\phi {\left(298\;K\right)}_{\Pi =0}}{\phi }$$

where *ϕ*(298 *K*)_*Π*=0_ is the resulting polymer volume fraction at null osmotic pressure at 298 K.

The same set of parameters used to fit the behavior of *R_H_* in water as a function of temperature could reproduce, within the experimental errors, the behavior of the external osmotic pressure as a function of the microgel volume ratio (*V*/*V_Max_*) for values higher than about 0.2 (see curves of Figure 7), at constant temperature of 298 K.

The value of polymer volume fraction obtained by the fitting curves at 298 K and *Π_EXT_* = 0, namely *ϕ*(298 K)*_Π_*_=0_, is 0.015, in good agreement with the experimental value (Table 1). Results of Figure 6 and Figure 7 suggest that in CS microgels, the degree of crosslinking is so small that the differences in the elastic contributions of CS_05 and CS_01 are below the experimental and fitting precision. Although there is ample variability of the value of A for PNIPAM microgels in the literature [42], the A value in Table 1 has the same order of magnitude of those obtained for microgels at a low degree of crosslinking [38].

Similarly, we investigated the size change of DC microgels in response to temperature and osmotic stress. The behavior of the hydrodynamic radius of DC_05 and DC_01 microgels in water as a function of temperature across the VPT is reported in Figure 8 (data reported in Table S2, Supplementary Materials), and the correlation between the osmotic pressure of the external solution and the shrinking ratio (*V*/*V_max_*) at 298 K for these microgels is illustrated in Figure 9 (data reported in Table S3, Supplementary Materials).

The extent of shrinkage across the VPT, defined as the ratio of microgel volume at 40 °C and 25 °C, was 0.007± 0.005 and 0.021 ± 0.009 for DC_05 and DC_01 microgels, respectively. By considering the corresponding values of CS microgels, we can notice that, among the tested microgel types, DC_05 microgel has the greatest size responsivity to temperature in water. The maximum microgel response to the osmotic stress at 298 K can be quantified by the minimum value of the shrinking ratio, which is 0.020 ± 0.006 and 0.04 ± 0.02 for DC_05 and DC_01 microgels, respectively (Figure 9), similarly to what detected for CS microgels (see Table S3, Supplementary Materials). However, the behavior of DC_05 microgel upon osmotic stress is different from that of all other investigated microgels, since small shrinking ratios are accessed with low values of external osmotic pressure. Therefore, DC_05 microgel overall displays the highest plasticity, i.e., capability to reduce its size both for an increase of temperature above the VPTT and as a consequence of an osmotic stress. A qualitative scale of microgels plasticity can be attempted on the basis of the osmotic stress experiments on the microgels with different morphologies: DC_05 > DC_01 > CS_05 ≳ CS_01. This relationship will be a guide in the study of the phagocytotic response of cells to the presence of the microgels.

We applied the Flory–Rehner theory to describe the behaviors of Figure 8 and Figure 9, in analogy to the analysis performed for CS microgels. The size dependence of DC_01 microgels on temperature and osmotic pressure could be qualitatively modeled, as shown by fitting curves of Figure 8b and Figure 9b, with a common set of parameters as reported in Table 2. However, A, C and D parameters for DC_01 microgels are different from those found for CS microparticles, thus reflecting the different architecture of DC and CS microgels. The degree of crosslinking from fit (*f*) was 0.003 and polymer volume fraction at 298 K for *Π_ext_* = 0 was 0.02, both of them slightly higher than the corresponding experimental values, equal to 0.001 and 0.012, respectively. It should be pointed out that none of the morphological details of the investigated microgels were taken into account in the Flory–Rehner model. Moreover, the degree of crosslinking (*f*) in the fitting equation is a parameter related to an “average” feature of the microgels, a quite unrealistic description of the effective microgel architectures where the crosslinks are confined in the microparticles domains.

On the basis of the response to the osmotic stress, we were able to define a scale of rigidity of the microgels with same composition but different crosslink distributions. A unified description of the behavior against temperature and osmotic stress stimuli was accomplished with the simple Flory–Rehner approach for CS_05, CS_01 and DC_01 morphologies. For DC_05 microgel, the *Π_ext_* vs. *V*/*V_max_* behavior at 298 K is qualitatively described with the same set of Flory–Rehner parameters used for DC_01 microgels (see Figure 9a and Table 2). However, the temperature behavior of DC_05 required a different set of fitting parameters, not compatible with the osmotic pressure response (Table 2). The qualitative unified description of the thermal and osmotic pressure response of topologically complex microgels, such as DC_05 microgel, is an ambitious task for any gel equation of state. The description of the response to osmotic stress is less efficient above the *V*/*V_Max_* transition for all systems, with an overestimation of the osmotic stress at *V*/*V_Max_* < 0.2, probably attributable to the chosen reference state at maximum swelling. The use of Flory–Rehner model, notwithstanding its great flexibility and overall simplicity, suggests that some fundamental feature of the investigated system is missing, such as the uneven distribution of crosslinks of these hybrid morphologies. Although the Flory–Rehner is still an invaluable guide for the understanding of the gel swelling [43], a unified description of the response to temperature and osmotic stress needs computationally more demanding models.

## 3. Conclusions

With the careful use of classic polymerization methods, such as temperature controlled surfactant-free precipitation polymerization, we were able to synthesize different dual-domain PNIPAM microgels, but maintaining the same chemical composition, with two different very low degrees of crosslinking. Hydrogel microparticles were characterized in terms of morphology, degree of hydration and thermal/osmotic stress behavior. While the water affinity at room temperature is quite insensitive to the difference of architecture and crosslink amount, the plasticity of DC_05 microgel, having the highest networking of the core and a polymer chain grafted shell, is significantly larger as compared to that of the other investigated microparticles. This information can guide the selection of microgels suitable to targeted drug delivery, since a higher plasticity inhibits the phagocytosis processes of particles interacting with the surface of cells [44]. Moreover, we showed that a unified description of the size behavior of microgels under thermal and osmotic perturbation is possible within a Flory–Rehner modified model, at least for not highly inhomogeneous architectures.

## 4. Materials and Methods

### 4.1. Materials

N-isopropylacrylamide, ≥99%, (NIPAM), N,N’-methylenebisacrylamide (BIS), potassium persulfate (KPS), fluorescein isothiocyanate isomer I (FITC), fluorescein O-methacrylate (Fluo-MA) (see Figure S1 of Supporting Material), poly(ethylene glycol) average *M_n_* 4000 Da, (PEG), dimethyl sulfoxide (DMSO), sodium carbonate and sodium bicarbonate were purchased from Sigma-Aldrich (Merck Life Science S.r.l., Milan, Italy). Methacryloxyethyl thiocarbamoyl rhodamine B (Rhod-MA) (see Figure S2 of Supporting Material) was purchased from Polysciences (Polysciences Europe GmbH, Hirschberg, Germany). All products were reagent grade and used as received. Water was Milli-Q purity grade (18.2 MΩ cm), produced with a deionization–ultrafiltration apparatus (Elga Pure-Lab Classic, ELGA LabWater, High Wycombe, UK) and filtered prior to use (nylon syringe filter, 0.20 μm pore size).

### 4.2. Preparation of PNIPAM Microgels with Un-Crosslinked Core and Crosslinked Shell (CS)

Typically, 0.3 g of NIPAM was dissolved in 24 mL deionized water. The solution was then poured into a 100 mL three-neck round bottom flask and purged with nitrogen gas under stirring for 1 h in an oil bath at 40 °C in order to remove dissolved oxygen. Then, 0.0275 g of KPS in 1 mL deionized water was added to the reaction vessel with a syringe to trigger the polymerization. As soon as the solution started to turn turbid (typically within 15 min), the temperature was raised to 70 °C for 1 h. Then 6 mL of deionized water containing BIS and 0.3 g of NIPAM was added to the reaction mixture for 1 h, using a syringe pump (0.1 mL/min). The reaction mixture was further stirred for 2 h at 70 °C. Finally, the nitrogen flow and the temperature controller were stopped and the microgel suspension was stirred overnight. Following this procedure, PNIPAM-based CS type microgel with two degrees of crosslinking were synthesized, using a % feed molar ratio of BIS to total amount of NIPAM of 0.5% mol/mol (CS_05) and 0.1% mol/mol (CS_01).

### 4.3. Preparation of PNIPAM Microgels with Crosslinked Core and Un-Crosslinked Shell (DC)

First, 0.3 g of NIPAM and BIS were dissolved in 24 mL deionized water and transferred in a three-neck round bottom flask. The solution was then purged with nitrogen under stirring in an oil bath at 40 °C for 1 h in order to remove oxygen. Then, 0.0275 g of KPS in 1 mL deionized water was added to the reaction vessel with a syringe to trigger the polymerization. As soon as the solution started to turn turbid (typically within 15 min), the temperature was raised to 70 °C for 1 h. Then, 6 mL of deionized water containing 0.3 g of NIPAM was added to the reaction mixture for 1 h using a syringe pump (0.1 mL/min). The reaction mixture was further stirred for 2 h at 70 °C. Finally, the nitrogen flow and temperature controller were stopped, and the microgel suspension was stirred overnight. Following this procedure, PNIPAM-based DC-type microgels with two degrees of crosslinking were prepared, using a % feed molar ratio of BIS to total amount of NIPAM of 0.5% mol/mol (DC_05) and 0.1% mol/mol (DC_01).

### 4.4. Purification of PNIPAM Microgels

The PNIPAM microgels were purified by centrifugation (10,000 rpm, 12 min/centrifugation round, at 40 °C) in order to remove unreacted monomers, oligomer chains and unreacted initiator molecules. Then, microgels were resuspended in deionized water, frozen with liquid nitrogen and lyophilized (freeze dryer Lio 5P, Cinquepascal, Milan, Italy). The microgels were stored as dry powder in a desiccator.

### 4.5. Preparation of Fluorescent PNIPAM Microgels

Fluorescent PNIPAM microgels with both morphologies, CS and DC, were synthesized by using a % feed molar ration of BIS to total amount of NIPAM of 0.5% mol/mol.

In order to prepare fluorescent CS_05 microgels, 0.3 g of NIPAM was dissolved in 24 mL of deionized water in a three-neck round bottom flask and added with a Fluo-MA (stock solution: 0.06 M in DMSO) to reach a feed molar ratio of Fluo-MA to NIPAM monomer of 0.008% mol/mol. The solution was purged with nitrogen under stirring in an oil bath at 40 °C for 1 h, in order to remove dissolved oxygen. Then, 0.0275 g of KPS in 1 mL deionized water was added to the reaction vessel with a syringe to initiate the polymerization. As soon as the solution started to turn turbid (typically within 5 min), the temperature was raised to 60 °C in 30 min. Then 6 mL of deionized water containing BIS, 0.3 g of NIPAM and Rod-MA (stock solution: 0.08 M in DMSO, to yield feed molar ratio of Rhod-MA to NIPAM of 0.003% mol/mol) was added using a syringe pump (0.3 mL/min) for 20 min.

For the preparation of fluorescent DC_05 microgels, 0.3 g of NIPAM, BIS and Rhod-MA (% feed molar ratio of Rhod-MA to NIPAM of 0.003% mol/mol) were dissolved in 24 mL of deionized water in a three-neck round bottom flask and the solution was purged with nitrogen under stirring in oil bath at 40 °C for 1 h in order to remove dissolved oxygen. Then, 0.0275 g of KPS in 1 mL deionized water was added to the reaction vessel with a syringe to trigger the polymerization. As soon as the solution started to turn turbid (typically within 5 min), the temperature was raised to 60 °C in 30 min. Then, 6 mL of deionized water containing 0.3 g of NIPAM and Fluo-MA (% feed molar ratio of Fluo-MA to NIPAM of 0.008% mol/mol) was added to the reaction mixture for 20 min using a syringe pump (0.3 mL/min).

Both types of fluorescent microgels were rapidly cooled under stirring in an ice bath and purified by centrifugation (14,000 rpm, 12 min/centrifugation round, 25 °C). Fluorescent microgels were then resuspended in deionized water, frozen with liquid nitrogen and lyophilized. The microgels were stored in the dark as dry powder in a desiccator.

### 4.6. Characterization of PNIPAM Microgels by Optical, Confocal Microscopy

PNIPAM microgels, in water suspension and at room temperature, were visualized with a differential interference contrast (DIC) filter under an optical microscope (Nikon Inverted Microscope Eclipse Ti-E, Nikon Instruments S.p.a, Florence, Italy) in transmission mode, by using a Plan Fluor 100.0x/1.30/0.20 oil immersion DIC H objective (Nikon), a DIC prism N2 (Nikon) and a Spectra Physics Ar^+^ ion laser (488 nm) as a light source. Fluorescent CS_05 and DC_05 microgels were characterized by confocal laser scanning microscopy (CLSM) by using the Nikon Inverted Microscope Eclipse Ti-E in confocal mode. The fluorescent probes covalently included in the microgels structures, Fluo-MA and Rhod-MA, were excited by the Ar^+^ ion laser (488 nm) (Spectra Physics, Santa Clara, CA, USA) and a Melles–Griot He–Ne laser (543 nm) (Lastek Pty Ltd, Thebarton, Australia), respectively. All images were captured using a Plan Fluor 100.0x/1.30/0.20 oil immersion objective and processed by using the EZ-C1 software (version 3.9, Nikon) and Image J freeware [45]. Fluorescent microgels were visualized in water suspension at room temperature, in the swollen state and in a 19.2% *w*/*v* PEG solution in the collapsed state under osmotic pressure application.

### 4.7. FITC Labeling of PEG (FITC-PEG)

First, 1 mL of fresh prepared FITC 5 mg/mL in DMSO was added dropwise to 10 mL PEG 5 mg/mL in carbonate buffer 0.1 M, pH 9. The reaction solution was stirred overnight in the dark. The polymer solution was then poured into a dialysis bag and extensively dialyzed in the dark against deionized water (benzoylated dialysis tubing with molecular weight cutoff (MWCO) of 2000 Da Sigma-Aldrich, Merck Life Science S.r.l., Milan, Italy) in order to remove unreacted FITC. The FITC-PEG solution was then frozen with liquid nitrogen and lyophilized. The polymer was stored in the dark as dry powder in a desiccator.

### 4.8. Determination of the Equilibrium Water Content of the PNIPAM Microgels: Maximum Swelling Degree

The equilibrium water content of PNIPAM microgels at 25 °C was determined according to a method described by Stenekes and Hennink [31,32]. Then, 3 mg of lyophilized PNIPAM microgels was equilibrated overnight with the aid of vortex agitation in 0.5 mL FITC-PEG 2 μg/mL. Due to absorption of water by the dry microgels, the FITC-PEG concentration increased. The hydrated microgels were separated from the solution by centrifugation (12,000 rpm, 10 min, 25 °C) and the increase in FITC-PEG concentration in the supernatant was quantified by spectrofluorimetry with a Spark^®^ (Tecan Milan, Italy) multimode microplate reader (96-well plate, λ_ex_ 470 nm–λ_em_ 519 nm, excitation and emission bandwidth 20 nm, gain 50, fixed z-position; the same parameters were used for the calibration in the linear range of 0–5 μg/mL, R^2^ = 0.9933).

### 4.9. Dynamic Light Scattering (DLS)

The average hydrodynamic radius (*R_H_*) of PNIPAM microgels was evaluated as a function of temperature, from 25 °C to 45 °C, and as a function of applied osmotic pressure, up to 1.8 atm, at 25 °C. The measurements were carried out with a dynamic light scattering at 90° using a BI-200SM Goniometer (Brookhaven Instruments Co., Holtsville, NY, USA) equipped with a solid state laser source at 532 nm and a BI-9000AT correlation board. Temperature was controlled with external unit circulating thermostated water in a coil placed in the vat containing the refractive index matching liquid, decaline. Autocorrelation functions, g^2^ (*q*, *t*), of the scattered intensity were analyzed by the CONTIN algorithm included in the standard software package of the instrument. For the evaluation of average radius as a function of temperature, dried microgel were equilibrated overnight in deionized water at a concentration of 3 mg/mL. At each temperature set point, the samples were thermally equilibrated for 15 min before analysis. For the measurements carried out in the presence of the osmotic pressure at room temperature, the dried PNIPAM microgels were equilibrated overnight in deionized water at a concentration of 6 mg/mL. PEG, *M_n_* 4000 (stock solution 500 mg/mL) was used as osmotically active polymer and was added stepwise to the microgels samples. The osmotic pressure at each given PEG concentration, was calculated assuming the PEG solutions as ideal according to

(12) $$\pi =\frac{RTC}{M_{n}}$$

where *R* is the universal gas constant, *C* is the polymer concentration (g/L) and *M_n_* is the average number molecular weight of the polymer. As *R_H_* is derived from the Einstein–Smoluchowski equation, the knowledge of the medium viscosity as a function of each PEG concentration used for the measurements of the size under osmotic stress is needed. To this aim, the viscosity values of PEG solutions at 25 °C and at the concentrations of 5.8, 12.3, 19.2 and 29.5% *w*/*v* were determined by an AR 2000 rheometer (TA Instruments, Milan, Italy). The values of viscosity for PEG solutions within the concentration range of 5.8–29.5% *w*/*v* were calculated by linear interpolation (R^2^ = 0.9945, see Figure S3, Supplementary Materials) and resulted in good agreement with the values reported in the literature [46,47]. Table 3 reports the values of viscosity of PEG solutions used for the evaluation of average hydrodynamic radius of PNIPAM microgels as a function of applied osmotic pressure by DLS analysis. The values of viscosity for PEG concentrations lower than 5.8% *w*/*v* were from the literature [46].

The reversibility of all types of PNIPAM microgels in response to temperature was also tested. Microgels were cooled down stepwise from 45 °C to 25 °C in order to study the reversible temperature response (see data in Table S2, Supplementary Materials). The reversibility of the response to osmotic stress is illustrated in Table S1, Supplementary Materials.

## Figures and Tables

**Table 1:** Swelling degree of PNIPAM microgels at 25 °C and corresponding polymer volume fraction.

	CS_05	CS_01	DC_05	DC_01
**% H_2_O (*w*/*w*)**	98.9 ± 3.0	98.6 ± 3.0	98.9 ± 3.0	98.7 ± 3.0
***ϕ***	0.010 ± 0.002	0.013 ± 0.003	0.010 ± 0.002	0.012 ± 0.002

**Table 2:** Parameters of the Flory–Rehner model describing the dependence of CS and DC microgel size on temperature and osmotic stress.

Microgel	*A*	*θ* (K)	*C*	*D*	*f*	*ϕ*_0_	*ϕ_collapsed_*	*ϕ* (298 K)_*Π*=0_
CS_05	−4.5	303.6	0.16	0.15	0.005	0.019	0.50	0.015
CS_01	−4.5	303.6	0.16	0.15	0.005	0.019	0.35	0.015
DC_05 ^1^	−12	304.6	0.10	0.10	0.010	0.008	0.99	0.0075
DC_05 ^2^	−0.88	303.6	0.313	0.333	0.003	0.019	- ^3^	0.0075
DC_01	–0.88	303.6	0.313	0.333	0.003	0.019	0.80	0.023

^1^*R_H_ vs* temperature fit parameters. ^2^ *Π_ext_ vs V/V_max_* at 298 K fit parameters. ^3^ The parameter *ϕ_collapsed_* is not used in the osmotic pressure fit.

**Table 3:** Concentration values and corresponding osmotic pressure and viscosity values of PEG solutions at 25 °C [46,47] used in DLS experiments for the evaluation of average hydrodynamic radius under applied osmotic pressure.

PEG % (*w*/*v*)	π (Atm)	Viscosity (cP)
0	0	0.89 ± 0.01
0.8	0.05	1.04 ± 0.05
2	0.13	1.23 ± 0.06
2.7	0.17	1.33 ± 0.06
3.3	0.20	1.43 ± 0.07
4.3	0.26	1.63 ± 0.08
5.8	0.36	1.89 ± 0.09 *
7.3	0.45	2.25 ± 0.11
9	0.55	2.70 ± 0.14
10	0.62	2.89 ± 0.14
12.3	0.75	3.49 ± 0.17 *
16.4	1	5.12 ± 0.26
19.2	1.2	6.24 ± 0.31 *
29.5	1.8	9.70 ± 0.49 *

* experimentally measured viscosity values by AR 2000 rheometer.

## Supplementary Materials

The following are available online at https://www.mdpi.com/2310-2861/6/4/34/s1, Table S1: Average hydrodynamic diameter (*d_H_*) of PNIPAM microgels in deionized water (*Π* = 0 atm), in the presence of PEG (*Π* = 0.36 atm) and after PEG removal by centrifugation (*Π* = 0 atm) and replacement of supernatant with fresh deionized water; temperature = 25 °C; data from DLS measurements, Table S2: Average hydrodynamic radius (*R_H_*) of PNIPAM microgels as a function of temperature, from 25 °C to 45 °C: data from DLS measurements (see manuscript text for the experimental part: Section 4.9), Table S3: Osmotic pressure of the external solution (*Π_EXT_*) and respective shrinking ratio (*V*/*V_max_*) at 25 °C for PNIPAM microgels: data from DLS measurements (see manuscript text for the experimental part: Section 4.9). Figure S1 and Figure S2: Structures of the fluorescent probes incorporated in the microgels. Figure S3: Measured PEG aqueous viscosities at 25 °C (AR 2000 rheometer TA Instruments, Milan, Italy).

## References

[1] Stuart M.A.C., Huck W.T., Genzer J., Müller M., Ober C., Stamm M., Sukhorukov G.B., Szleifer I., Tsukruk V.V., Urban M. (2010). Emerging applications of stimuli-responsive polymer materials. Nat. Mater..

[2] Agrawal G., Agrawal R. (2018). Functional microgels: Recent advances in their biomedical applications. Small.

[3] Karg M., Pich A., Hellweg T., Hoare T., Lyon L.A., Crassous J.J., Suzuki D., Gumerov R.A., Schneider S., Potemkin I.I. (2019). Nanogels and microgels: From model colloids to applications, recent developments, and future trends. Langmuir.

[4] Fernandez-Nieves A., Wyss H., Mattsson J., Weitz D.A. (2011). Microgel Suspensions: Fundamentals and Applications.

[5] Bai H., Sheng K., Zhang P., Li C., Shi G. (2011). Graphene oxide/conducting polymer composite hydrogels. J. Mater. Chem..

[6] Virtanen O., Mourran A., Pinard P., Richtering W. (2016). Persulfate initiated ultra-low cross-linked poly (*N*-isopropylacrylamide) microgels possess an unusual inverted cross-linking structure. Soft Matter.

[7] Mueller E., Alsop R.J., Scotti A., Bleuel M., Rheinstädter M.C., Richtering W., Hoare T. (2018). Dynamically cross-linked self-assembled thermoresponsive microgels with homogeneous internal structures. Langmuir.

[8] Varga I., Gilányi T., Meszaros R., Filipcsei G., Zrinyi M. (2001). Effect of cross-link density on the internal structure of poly (*N*-isopropylacrylamide) microgels. J. Phys. Chem. B.

[9] Aderem A., Underhill D.M. (1999). Mechanisms of phagocytosis in macrophages. Annu. Rev. Immunol..

[10] Sierra-Martín B., Laporte Y., South A.B., Lyon L.A., Fernández-Nieves A. (2011). Bulk modulus of poly(*N*-isopropylacrylamide) microgels through the swelling transition. Phys. Rev. E.

[11] Sierra-Martin B., Frederick J.A., Laporte Y., Markou G., Lietor-Santos J.J., Fernandez-Nieves A. (2011). Determination of the bulk modulus of microgel particles. Colloid Polym. Sci..

[12] Guo M., Wyss H.M. (2011). Micromechanics of Soft Particles. Macromol. Mater. Eng..

[13] Cerroni B., Pasale S.K., Mateescu A., Domenici F., Oddo L., Bordi F., Paradossi G. (2015). Temperature-Tunable Nanoparticles for Selective Biointerface. Biomacromolecules.

[14] Pasale S.K., Cerroni B., Ghugare S., Paradossi G. (2014). Multiresponsive Hyaluronan-p(NiPAAm) “Click”-Linked Hydrogels. Macromol. Biosci..

[15] Pelton R.H., Chibante P. (1986). Preparation of aqueous latices with *N*-isopropylacrylamide. Colloids Surf..

[16] Su W., Zhao K., Wei J., Ngai T. (2014). Dielectric Relaxations of poly(*N*-isopropylacrylamide) Microgels Near the Volume Phase Transition Temperature: Impact of Cross-Linking Density Distribution on the Volume Phase Transition. Soft Matter.

[17] Gelissen A.P.H., Oppermann A., Caumanns T., Hebbeker P., Turnhoff S.K., Tiwari R., Eisold S., Simon U., Lu Y., Mayer J. (2016). 3D Structures of Responsive Nanocompartmentalized Microgels. Nano Lett..

[18] Siemes E., Nevskyi O., Sysoiev D., Turnhoff S.K., Oppermann A., Huhn T., Richtering W., Wöll D. (2018). Nanoscopic Visualization of Cross-Linking Density in Polymer Networks with Diarylethene Photoswitches. Angew. Chem. Int. Ed. Engl..

[19] Karanastasis A.A., Zhang Y., Kenath G.S., Lessard M.D., Bewersdorf J., Ullal C.K. (2018). 3D mapping of nanoscale crosslink heterogeneities in microgels. Mater. Horiz..

[20] Scheffold F. (2020). Pathways and challenges towards a complete characterization of microgels. Nat. Commun..

[21] Alvarez L.H., Eisold S., Gumerov R.S., Strauch M., Rudov A.A., Lenssen P., Merhof D., Potemkin I.I., Simon U., Wöll D. (2019). Deformation of Microgels at Solid–Liquid Interfaces Visualized in Three-Dimension. Nano Lett..

[22] Domenici F., Brasili F., Oddo L., Cerroni B., Bedini A., Bordi F., Paradossi G. (2019). Long-term physical evolution of an elastomeric ultrasound contrast microbubble. J. Colloid Interf. Sci..

[23] Garini Y., Vermolen B.J., Young I.T. (2005). From micro to nano: Recent advances in high-resolution microscopy. Curr. Opin. Biotechnol..

[24] Wu X., Pelton R.H., Hamielec A.E., Woods D.R., McPhee W. (1994). The kinetics of poly(*N*-isopropylacrylamide) microgel latex formation. Colloid Polym. Sci..

[25] Stieger M., Richtering W., Pedersen J.S., Lindner P. (2004). Small-angle neutron scattering study of structural changes in temperature sensitive microgel colloids. J. Chem. Phys..

[26] Kesenci K., Piskin E. (1998). Production of poly[(ethylene glycol dimethacrylate)-co-acrylamide] based hydrogel beads by suspension copolymerization. Macromol. Chem. Phys..

[27] Shukla P.G., Rajagopalan N., Bhaskar C., Sivaram S. (1991). Cross-linked starch-urea formaldehyde (St-UF) as a hydrophilic matrix for encapsulation: Studied is swelling and release of carbofuran. J. Control. Release.

[28] Murat Elçin Y. (1995). Encapsulation of urease enzyme in xanthan-alginate spheres. Biomaterials.

[29] Atkins T.W., McCallion R.L., Tighe B.J. (1993). Incorporation and release of fluorescein isothiocyanate-linked dextrans from a bead-formed macroporous hydrophilic matrix with potential for sustained release. Biomaterials.

[30] Wang N., Wu X.S. (1998). A novel approach to stabilization of protein drugs in poly(lactic-co-glycolic acid) microspheres using agarose hydrogel. Int. J. Pharm..

[31] Stenekes R.J., Hennink W.E. (1999). Equilibrium Water Content of Microspheres Based on Cross-Linked Dextran. Int. J. Pharm..

[32] Paradossi G., Cavalieri F., Chiessi E. (2005). Proton Fluctuations and Water Diffusion in Dextran Chemical Hydrogels Studied by Incoherent Elastic and Quasielastic Neutron Scattering. Carbohydr. Res..

[33] Yanwei Ding Y., Zhang G. (2007). Collapse and Aggregation of Poly(*N*-isopropylacrylamide) Chains in Aqueous Solutions Crowded by Polyethylene Glycol. J. Phys. Chem. C.

[34] Aangenendt F.J., Mattsson J., Ellenbroek W.G., Wyss H.M. (2017). Mechanics from Calorimetry: Probing the Elasticity of Responsive Hydrogels. Phys. Rev. Appl..

[35] Flory P.J. (1953). Principles of Polymer Chemistry.

[36] Paradossi G., Chiessi E. (2017). Tacticity-Dependent Interchain Interactions of Poly(*N*-Isopropylacrylamide) in Water: Toward the Molecular Dynamics Simulation of a Thermoresponsive Microgel. Gels.

[37] Chiessi E., Paradossi G. (2016). Influence of Tacticity on Hydrophobicity of Poly(*N*-isopropylacrylamide): A Single Chain Molecular Dynamics Simulation Study. J. Phys. Chem. B.

[38] Lopez G.C., Richtering W. (2017). Does Flory–Rehner Theory Quantitatively Describe the Swelling of Thermoresponsive Microgels?. Soft Matter.

[39] Routh A.F., Fernandez-Nieves A., Bradley M., Vincent B. (2006). Effect of Added Free Polymer on the Swelling of Neutral Microgel Particles:  A Thermodynamic Approach. J. Phys. Chem. B.

[40] Saunders B.R., Vincent B. (1997). Osmotic de-swelling of polystyrene microgel particles. Colloid Polym Sci..

[41] Saunders B.R., Vincent B. (1996). Thermal and osmotic deswelling of poly(NIPAM) microgel particles. J. Chem. Soc. Faraday Trans..

[42] Fernández-Barbero A., Fernández-Nieves A., Grillo I., López-Cabarcos E. (2002). Structural modifications in the swelling of inhomogeneous microgels by light and neutron scattering. Phys. Rev. E.

[43] Sbeih S., Mohanty P.S., Morrow M.R., Yethiraj A. (2019). Structural parameters of soft PNIPAM microgel particles as a function of crosslink density. J. Colloid Interf. Sci..

[44] Anselmo A.C., Zhang M., Kumar S., Vogus D.R., Menegatti S., Helgeson M.E., Mitragotri S. (2015). Elasticity of Nanoparticles Influences Their Blood Circulation, Phagocytosis, Endocytosis, and Targeting. ACS Nano.

[45] ImageJ Image Processing and Analysis in Java. https://imagej.net/Fiji.html#Downloads.

[46] Sim S.L., He T., Tscheliessnig A., Mueller M., Tan R.B.H., Jungbauer A. (2012). Branched Polyethylene Glycol for Protein Precipitation. Biotechnol. Bioeng..

[47] Rubinson K.A., Meuse C.W. (2013). Deep hydration: Poly(ethylene glycol) Mw 2000–8000 Da probed by vibrational spectrometry and small-angle neutron scattering and assignment of *Δ*G° to individual water layers. Polymer.

