# Supplementary material for: Microgel Particles with Distinct Morphologies and Common Chemical Compositions: A Unified Description of the Responsivity to Temperature and Osmotic Stress
Source: Gels. 2020 Oct 16;6(4):34. doi: 10.3390/gels6040034 (PMC7709680; doi:10.3390/gels6040034)
Supplement: Supplementary file 1 [file gels-06-00034-s001.pdf]

gels

Supplementary Materials

Microgel Particles with Distinct Morphologies and Common Chemical Compositions: a Unified Description of the Responsivity to Temperature and Osmotic Stress

Andrea Ruscito, Ester Chiessi, YosraToumia, Letizia Oddo, Fabio Domenici and Gaio Paradossi*

1. Chemical Structures of the Dyes Used for the Labelling of PNIPAM Microgels.

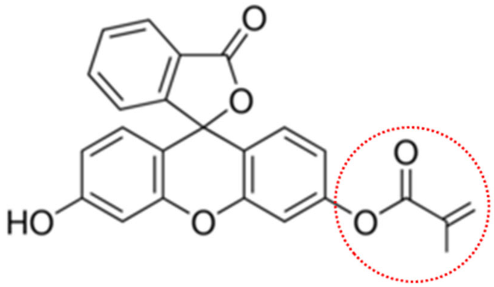

Figure S1. Chemical structure of fluorescein O-methacrylate (Fluo-MA): the red dotted ring highlights the (meth) acrylate moiety of the molecule exploited for the polymerization.

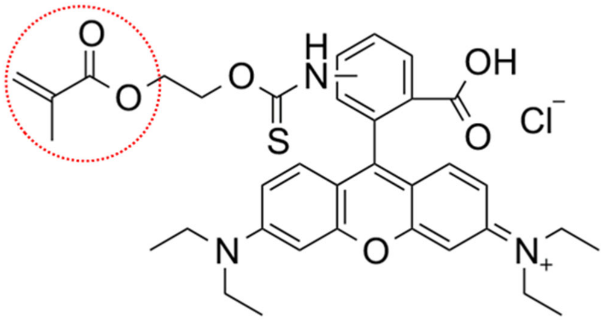

Figure S2. Chemical structure of methacryloxyethyl thiocarbamoyl rhodamine B (Rhod-MA): the red dotted ring highlights the (meth)acrylate moiety of the molecule exploited for the polymerization.

2. Testing the Reversibility of the Osmotic Effect on PNIPAM Microgels

The reversibility of all types of poly(N-isopropylacrylamide) (PNIPAM) microgels (CS_05, CS_01, DC_05, DC_01) upon osmotic stress removal was probed at 25 °C. To this aim, polyethyleneglycol (PEG), Mn 4000 (stock solution 500 mg/mL) was used as osmotically active polymer and was added to the microgels aqueous suspensions at a concentration of 5.8 % w/v, corresponding to an applied osmotic pressure of 0.36 atm. The average hydrodynamic diameter of microgel particles was monitored by dynamic light scattering (DLS) before PEG addition, upon addition of PEG and after PEG removal by centrifugation (12,000 rpm, 10 min, 2 rounds, 25 °C) and replacement of fresh deionized water, as reported in Table S1.

Table S1. Average hydrodynamic diameter (d_h) of PNIPAM microgels in deionized water (Π = 0 atm), in the presence of PEG (Π = 0.36 atm) and after PEG removal by centrifugation (Π = 0 atm) and replacement of supernatant with fresh deionized water; temperature = 25 °C; data from DLS measurements.

	d_h (nm), Π = 0 (Atm)	d_h (nm), Π = 0.36 (Atm)	d_h (nm), Washing, Π = 0 (Atm)
**CS_05**	3100 ± 300	2100 ± 300	3000 ± 300
**CS_01**	3400 ± 200	2200 ± 200	3100 ± 300
**DC_05**	3400 ± 300	1700 ± 100	3200 ± 300
**DC_01**	3400 ± 300	1900 ± 200	3100 ± 300

3. Dependence of PNIPAM Microgel Size on Temperature

Table S2. Average hydrodynamic radius (R_h) of PNIPAM microgels as a function of temperature, from 25 °C to 45 °C: data from DLS measurements (see manuscript text for the experimental part: Section 4.9).

	CS_05		CS_01		DC_05		DC_01	
**Temp. (°C)**	R_h (nm) Heating	R_h (nm) Cooling	R_h (nm) Heating	R_h (nm) Cooling	R_h (nm) Heating	R_h (nm) Cooling	R_h (nm) Heating	R_h (nm) Cooling
25	1600 ± 140	1600 ± 150	1700 ± 170	1700 ± 50	1750 ± 200	1600 ± 50	1800 ± 160	1600 ± 150
28	1600 ± 140	1500 ± 40	1800 ± 200	1600 ± 250	1700± 160	1600 ± 160	1800 ± 150	1800 ± 150
32	870 ± 100	800 ± 100	840 ± 85	600 ± 10	1150 ± 100	1200 ± 100	887 ± 130	740 ± 30
35	530 ± 30	510 ± 50	700 ± 50	490 ± 5	450 ± 20	400 ± 13	560 ± 18	470 ± 60
40	500 ± 40.0	510 ± 30	600 ± 140	500 ± 30	340 ± 40	350 ± 10	500 ± 30	520 ± 30
45	500 ± 100	500 ± 100	630 ± 4.0	630 ± 4.0	330 ± 30	330 ± 30	500± 60	520 ± 60

4. Dependence of PNIPAM Microgel Size on Osmotic Stress

Table S3. Osmotic pressure of the external solution (Π_{EXT}) and respective shrinking ratio (V/V_{max}) at 25 °C for PNIPAM microgels: data from DLS measurements (see manuscript text for the experimental part: Section 4.9).

Π_{EXT} (Atm)	V/V_{max} (CS_05)	V/V_{max} (CS_01)	V/V_{max} (DC_05)	V/V_{max} (DC_01)
0	1.0 ± 0.6	1.0 ± 0.6	1.0 ± 0.6	1.0 ± 0.6
0.05	1.0 ± 0.6	1.0± 0.6	0.7 ± 0.3	0.8 ± 0.4
0.13	0.7 ± 0.6	0.43 ± 0.25	0.05 ± 0.03	0.5 ± 0.25
0.17	0.8 ± 0.4	0.47 ± 0.25	0.06 ± 0.03	0.3 ± 0.2
0.20	0.5 ± 0.3	0.4 ± 0.3	0.04 ± 0.02	0.3 ± 0.1
0.26	0.4 ± 0.3	0.3 ± 0.2	0.04± 0.02	0.2 ± 0.1
0.36	0.4 ± 0.2	0.4 ± 0.	0.04 ± 0.02	0.2 ± 0.1
0.45			0.03 ± 0.01	
0.55		0.4 ± 0.2	0.03 ± 0.02	0.2 ± 0.07
0.62	0.3 ± 0.2		0.03 ± 0.02	0.13 ± 0.06
0.75	0.16 ± 0.1	0.1 ± 0.1	0.02 ± 0.02	0.09 ± 0.05
1.0	0.02 ± 0.01	0.03 ± 0.02	0.02 ± 0.01	0.05 ± 0.02
1.2	0.03 ± 0.01	0.03 ± 0.02	0.02 ± 0.02	0.06 ± 0.03
1.8	0.03 ± 0.02	0.03 ± 0.02	0.03 ± 0.02	0.04 ± 0.02

5. PEG Aqueous Solution Viscosities at 25 °C

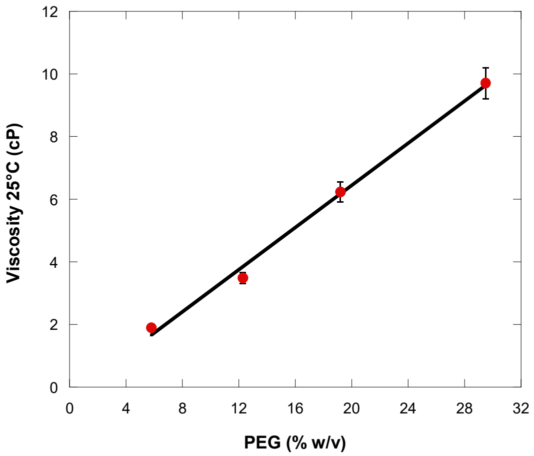

Figure S3. Measured PEG aqueous viscosities at 25 °C (AR 2000 rheometer TA Instruments, Milan, Italy).
